# Fasting Plasma Glucose, Self-Appraised Diet Quality and Depressive Symptoms: A US-Representative Cross-Sectional Study

**DOI:** 10.3390/nu9121330

**Published:** 2017-12-07

**Authors:** Erin Hoare, Sarah R. Dash, Pia Varsamis, Garry L. Jennings, Bronwyn A. Kingwell

**Affiliations:** 1 Metabolic and Vascular Physiology, Baker Heart and Diabetes Institute, 75 Commercial Rd., Melbourne, VIC 3004, Australia; Sarah.Dash@baker.edu.au (S.R.D.); Pia.Varsamis@baker.edu.au (P.V.); garry.jennings@sydney.edu.au (G.L.J.); Bronwyn.Kingwell@baker.edu.au (B.A.K.); 2 Sydney Medical School, University of Sydney, Sydney, NSW 2006, Australia

**Keywords:** fasting plasma glucose, depression, population health, lifestyle, diet quality

## Abstract

Depression and type 2 diabetes (T2D) contribute significantly to global burden of disease and often co-occur. Underpinning type 2 diabetes is poor glycaemic control and glucose is also an obligatory substrate for brain metabolism, with potential implications for cognition, motivation and mood. This research aimed to examine the relationships between fasting plasma glucose and depressive symptoms in a large, population representative sample of US adults, controlling for other demographic and lifestyle behavioural risk factors. Using the 2013–2014 National Health and Nutrition Examination Survey (NHANES) data, this study first investigated the relationship between fasting plasma glucose and mental disorders at a population-level, accounting for demographic, health behavioural and weight-related factors known to co-occur with both type 2 diabetes and mental disorders. Depressive symptoms were derived from the 9-item Patient Health Questionnaire. Fasting plasma glucose was obtained through medical examination and demographic (age, household income, sex) and health characteristics (perceived diet quality, daily time sedentary) were self-reported. Body mass index was calculated from objectively measured height and weight. In the univariate model, higher fasting plasma glucose was associated with greater depressive symptoms among females (*b* = 0.24, 95% CI = 0.05, 0.43, *p* < 0.05), but not males. In the final fully adjusted model, the relationship between fasting plasma glucose and depressive symptoms was non-significant for both males and females. Of all independent variables, self-appraised diet quality was strongly and significantly associated with depressive symptoms and this remained significant when individuals with diabetes were excluded. Although diet quality was self-reported based on individuals’ perceptions, these findings are consistent with a role for poor diet in the relationship between fasting plasma glucose and depressive symptoms.

## 1. Introduction

Depression and non-communicable diseases (NCDs) including type 2 diabetes (T2D), cardiovascular disease and obesity, are leading contributors to burden of disease and, are often comorbid [1]. The clinical, environmental and lifestyle antecedents for NCDs are widely recognised and subsequently promoted as key targets in prevention and treatment [2]. The possibility for shared underlying risk factors for the development and expression of common mental disorders (CMDs) such as anxiety and depression, particularly lifestyle related factors, has also been recently promoted [3]. For example, research demonstrates that although the determinants of mental health are complex, dietary patterns including high intake of energy, saturated fats and refined sugar, as well as low consumption of fruits and vegetables have been associated with risk and onset of depression [4,5,6]. Poor quality of diet is also a precursor to NCDs through mechanisms including overweight/obesity, high blood pressure, impaired glucose metabolism, and dyslipidaemia [7]. As such, understanding the relationship between mental and NCDs is of critical public health importance for primary and secondary prevention, and for treatment and management of individuals suffering such conditions.

The relationship between depression and T2D is of particular and current interest [8]. These disorders are often comorbid; individuals with T2D are at increased risk of developing depression and the reverse has also been shown to be true [9,10]. Underpinning type 2 diabetes is poor glycaemic control and glucose is also an obligatory substrate for brain metabolism. Poor glycaemic control may therefore also affect mood, fatigue and cognitive functioning [11,12]. Recent evidence suggests that glucose dysregulation is associated with a broad range of mental health conditions such as increased levels of depression, anxiety, stress-related problems, and psychological distress [13,14,15]. A meta-analysis [16] has shown glucose dysregulation as a potential mechanistic pathway in the link between depression and type 2 diabetes. However, studies often fail to examine factors which may provide insight into the relationship and which are known to affect glycaemic dysregulation and mental health such as diet, physical activity, and overweight/obesity. Gender differences have also been observed in epidemiological research to date. The recent Global Burden of Disease study revealed that smoking, high systolic blood pressure and high body mass index (BMI) emerged as the highest contributors to disability-adjusted life years for men, and high systolic blood pressure, high body mass index and high fasting plasma glucose were the leading contributing risk factors for women [17]. The investigation of potential gender specific risk factors for disease onset is of current and critical importance. 

While the potential for targeting joint risk factors for diabetes-related and mental health outcomes is appealing, the evidence to date is constrained by self-reported behavioural data within specific sub-groups (e.g., individuals suffering metabolic syndrome [18], middle to older aged adults [19], male war veterans [20]). As such, the goal of this research is to examine the relationships between fasting plasma glucose and depressive symptoms in a large, population representative sample of US adults. Importantly, the study will consider lifestyle behaviour factors known to co-occur with both depressive symptoms and poor glycaemic control including overweight/obesity, self-appraised diet quality, and daily time spent sedentary, examined separately for males and females. To our knowledge this is the first population-level examination of this kind, adopting a joint biomedical, psychosocial and lifestyle behavioural approach.

## 2. Materials and Methods

### 2.1. Study Design

This study investigated the 2013–2014 National Health and Nutrition Examination Survey (NHANES) data, which forms one component of a series of US population-level representative surveys [21]. The cross-sectional survey aims to capture the health and nutritional status of the US civilian, non-institutionalised population, and is led by the National Centre for Health Statistics and the Centres for Disease Control and Prevention. Data collection occurred during interviews within participants’ homes, and through medical examination and subsequent laboratory assessments in the Mobile Examination Centre (further details in sub-section below) [22]. The protocols for the conduct of NHANES were approved by the National Center for Health Statistics institutional review board (NCHS IRB/ERB), and informed consent was obtained from all participants (NCHS IRB/ERB protocols #2011-17). The methods, protocols and data collection tools are available at https://wwwn.cdc.gov/nchs/nhanes/ContinuousNhanes/Default.aspx?BeginYear = 2013 [23]. Ethics exemption was received for this study from Alfred Health, Melbourne, Australia.

### 2.2. Participants

A complex, multi-stage cluster sampling probability design was used to sample participants, with an oversample of groups. The purpose of oversampling was to increase reliability and precision of estimates of health status indicators for these sub-groups of particular public health interest (more information available [21]. In 2013–2014 there were 14,332 individuals selected for NHANES and of those selected, 10,175 (response rate 71%) completed the interview and 9813 (response rate 68.5%) participated in the medical examination. A total of 2145 participants had fasting plasma glucose measures and complete (laboratory, questionnaire and medical examination) data available and were subsequently included in this study. Further details pertaining to the demographic and health characteristics of this sample are reported in the results section.

### 2.3. Data Collection

The NHANES interviews were completed by trained interviewers in participants’ homes by using Computer-Assisted Personal Interview (CAPI) system. The interview includes survey questions on demographics (e.g., age, sex, race/ethnicity, education, marital status), general health (health conditions, body composition), and lifestyle variables (e.g., smoking, physical activity, diet, substance use). Additionally, anthropometric (height, weight) and laboratory analyses (e.g., nutritional biomarkers, markers of disease risk) were collected in the mobile examination centre (MEC). All samples were shipped to University of Missouri-Columbia for analysis. Complete information on laboratory procedures and analyses are available elsewhere. All information summarised here is taken from NHANES documentation, previously reported [22].

#### 2.3.1. Depressive Symptoms

The nine-item Patient Health Questionnaire (PHQ-9) was used to assess the presence and severity of depressive symptoms [24]. The tool was initially developed based on criteria from the Diagnostic and Statistical Manual of Mental Disorders, Fourth Edition (DSM-IV) and was one of the first questionnaires to assess symptoms based on clinical diagnostic criteria [25]. The PHQ-9 is used as a brief diagnostic and severity measure in both research and clinical practice [26]. Criterion validity has been established for diagnosing major depressive disorder in comparison to standard clinical diagnostic interview [27], and as an outcome measure to detect depression changes over time [28].

The PHQ-9 tool asks participants how often over the preceding two weeks they have experienced problems such as; ‘little interest or pleasure in doing things’, ‘feeling down, depressed or hopeless’, and ‘feeling tired or overeating’. Responses are scored 0–4, where 0 = not at all, and 4 = nearly every day and total possible scores range from 0 to 27. While diagnostic criteria for depressive disorders has been reported (e.g., scores > 10 indicate moderate to severe major depression), it was decided that responses would be reported as continuous to highlight the associations with symptom frequency and severity. Validity has been previously shown for continuous PHQ scores as a measure of depressive symptoms [26,29,30]. 

#### 2.3.2. Fasting Plasma Glucose

Blood samples were collected, processed and stored at the mobile examination centre (MEC), where participant fasting status (assigned to morning examination session and had fasted at least nine hours) was assessed prior to collection. There were seven exclusion criteria for participation including haemophilia and chemotherapy safety exclusions, fasting less than nine hours, or refusing phlebotomy. Fasting plasma glucose was measured in mg/dL, (converted into mmol/L for analysis in this study) and the lower detection limit for fasting glucose was 2.0 mg/dL. 

#### 2.3.3. Body Mass Index

Anthropometric measurements were collected in the MEC by trained health examiners using standardised protocols. Body weight (kg) was measured using a digital weight scale, and standing height (cm) using a stadiometer. Body Mass Index (BMI = kg/m^2^) was calculated as a continuous variable for all participants.

#### 2.3.4. Lifestyle Factors

Data on self-appraised healthfulness of diet was examined as part of the Diet Behaviour and Nutrition questionnaire, where participants were asked about their beliefs regarding the general quality of their diet, and participant responses to ‘in general, how healthy is your overall diet?’ were recorded on a scale of 1 (excellent) to 5 (poor). This measure has been validated against a more comprehensive dietary index in a US representative sample [31]. Self-appraised diet quality was merged as three levels; poor, good/fair, excellent/very good. Further, sedentary behaviour was assessed via the Physical Activity Questionnaire; participants were asked about total time (in minutes/day) spent sitting on a typical day, including at school, home, commuting, leisure activities (reading, playing cards, watching TV), time with friends, at a desk/computer, etc., not including time spent sleeping. Time spent sedentary was calculated as <3 h, 3–5 h, and more than five hours spent sedentary. 

#### 2.3.5. Demographics

Detailed data on individual, family and household level demographics were collected from all participants. Age at screening (years, further stratified as described in the results section), gender (male/female), and annual household income were all self-reported. Other demographic information were collected but excluded from the current study as considered not applicable. Age, gender and household income were selected as appropriate covariates given the onset of mental health problems is often age-dependent [32]. In addition, gender and relative socio-economic advantage and disadvantage differences are widely-reported in relation to the prevalence of mental disorders and associated symptomatology [33].

### 2.4. Statistical Analysis

Participant characteristics are reported as proportions and means (standard deviations). Sample sizes are unweighted, whereas proportions and means are weighted using linearized estimates to account for the sampling strategy and obtain population-representative findings. The fasting subsample weights were used in all analyses. Differences between males and females were investigated using regression models, and significance was assumed at *p* < 0.05. Linear regression models were completed to examine the relationship between depressive symptoms and fasting glucose independently (Model 1), then additionally adjusted for covariates age (stratified as indicated in Table 1) and household income (Model 2), weight status (Model 3), and lifestyle factors in self-reported healthiness of diet and average daily time spent sedentary (Model 4). Unstandardised coefficients and associated 95% confidence intervals and *p* values are reported, as are standardised beta (β) coefficients. Significance was assumed at *p* < 0.05. Further analyses were completed to examine the described relationships with individuals excluded who reported ‘yes’ to whether their doctor had diagnosed them with diabetes, henceforth referred to as individuals with diabetes (Supplementary Table S1). All data were analysed using Stata release V.14.1 (Stata Corp., College Station, TX, USA, 2013), and SPSS V.22 (IBM Corp., Released 2013, IBM SPSS Statistics for Windows, Version 22.0. IBM Corp., Armonk, NY, USA).

## 3. Results

Participant health and demographic characteristics are reported in Table 1, with proportions weighted to account for the sampling strategy. Females reported significantly (*p* < 0.05) greater levels of depressive symptoms (*m* = 3.6, SD = 4.4) compared to males (*m* = 2.5, SD = 3.8), in a possible score of 0–27. The proportion meeting cut-off for potential clinical depression (greater than or equal to 10 out of 27 [26]) was 10% among females and 6% among males. Fasting plasma glucose levels were significantly higher among males (*m* = 5.9, SD = 1.6) compared to females (*m* = 5.7, SD = 1.7) (*p* < 0.05). Average BMI was significantly higher among females (*m* = 29.7, SD = 8.2) compared to males (*m* = 28.5, SD = 6.2). Males and females were similar across all other demographic and health characteristics.

Regression findings are reported in Table 2. Higher fasting plasma glucose was associated with greater depressive symptoms among females in a univariate model (*b* = 0.24, 95% CI = 0.05, 0.43, *p* < 0.05) and this relationship remained significant when age and household income were included in the model (*b* = 0.21, 95% CI = 0.03–0.38, *p* < 0.05). When demographics were included, older age was significantly associated with increased depressive symptoms among males (*b* = 0.22, 95% CI = 0.04–0.39, *p* < 0.05) and higher household income was associated with lower levels of depressive symptoms for both males (*b* = −0.44, 95% CI = −0.64, −0.24, *p* < 0.05) and females (*b* = −0.44, 95% CI = −0.78–0.32, *p* < 0.05).

When BMI was additionally included in the model, the relationship between fasting plasma glucose and depressive symptoms among females became non-significant. The relationship between older age and increased depressive symptoms remained significant for males (*b* = 0.21, 95% CI = 0.04, 0.38, *p* < 0.05), and the relationship between higher household income and lower depressive symptoms was mostly unchanged for both males and females. Higher BMI was significantly associated with higher levels of depressive symptoms among females (*b* = 0.06, 95% CI = 0.02–0.09, *p* < 0.05), whereas this relationship was non-significant among males. 

In the final model, fully adjusted for age, household income, BMI, self-reported diet quality and daily time spent sedentary, the relationship between fasting plasma glucose and depressive symptoms was non-significant for both males and females. Of all independent variables, self-reported diet quality was strongly and significantly associated with depressive symptoms. Females who reported highest quality diet (very good/excellent) also experienced the lowest levels of depressive symptoms (*b* = −4.11, 95% CI = −5.64, −2.57, *p* < 0.05), compared to those who rated their diet as poor. Those females who reported their diet as good/fair also experienced lower depressive symptoms compared to those who self-reported their diet as poor (*b* = −3.31, 95% CI = −4.77, −1.86, *p* < 0.05). The same trend was observed among males whereby those rating their diet as very good/excellent experienced lower levels of depressive symptoms (*b* = −2.85, 95% CI = −4.58, −1.12, *p* < 0.05), as did those rating good/fair (*b* = −2.46, 95% CI = −4.14, −0.78, *p* < 0.05), compared to males who self-reported poor quality of diet. Older age remained significant for males in the relationship with depressive symptoms (*b* = 0.25, 95% CI = 0.07, 0.43, *p* < 0.05), as did higher household income for both males (*b* = −0.39, 95% CI = −0.57, −0.20, *p* < 0.05) and females (*b* = −0.44, 95% CI = −0.68, −0.21, *p* < 0.05).

Further analyses to examine the above relationships excluding individuals with diabetes, revealed a non-significant relationship between fasting plasma glucose and depressive symptoms among females (Supplementary Table S1). Other findings were consistent to those found in the full sample. 

## 4. Discussion

While the relationship between fasting plasma glucose and depressive symptoms was significant among females, this relationship was attenuated in the final fully adjusted model and non-significant in the model with diabetics excluded. Self-appraised healthier diet quality and higher household income were significantly related to lower levels of depressive symptoms for both males and females, and these findings held true when diabetics were excluded from analysis. Older age appeared to coincide with increased depressive symptoms among males, and increased BMI was associated to higher levels of depressive symptoms among females.

To our knowledge this was the first population-level examination of associations between fasting plasma glucose and depressive symptomatology that adopted a joint biomedical, psychosocial and health behavioural design. The sample is representative of the wider US population in terms of prevalence of depression [34], mean fasting plasma glucose [35], BMI [36], and average daily time spent sedentary [37]. A small (5%) proportion reported their diets to be ‘poor’. Recent epidemiological trends show the proportion of US adults with poor diets (categorised by foods linked to increased risk of cardiovascular and metabolic conditions) was approximately 45% [38] and it is likely that the proportion of participants consuming diets considered poor quality was under-reported. Higher levels of depression among females compared to males is unsurprising, with prevalence known to occur at higher rates among females [39]. Males, on average, had higher fasting plasma glucose than females and gender differences in fasting plasma glucose levels have been previously observed in the literature [40,41]. Overweight/obesity tends to be more prevalent among females than males, as previously shown among the US population [42].

Findings among this large, US-representative sample indicated a significant association between elevated fasting plasma glucose levels and heightened depressive symptoms among females. Clinical studies suggest both acute and sustained elevation in blood glucose levels may affect cerebral function and mental state through a variety of metabolic and vascular mechanisms. These include modulation of blood flow, insulin sensitivity and tissue glucose uptake [43] possibly leading to neurotransmitter imbalance [44] neuronal death and loss of memory [45]. Importantly, glucose has been shown to reduce hypothalamic blood flow and activity, which may ultimately impact motivation, reward processing, and other components of mental functioning [43]. Given known comorbidity of type 2 diabetes and mental disorders [9,10], findings here warrant monitoring of glucose levels and other biomarkers, in addition to psychological, lifestyle behavioural and weight factors, for type 2 diabetes and depression risk surveillance. The relationship between fasting plasma glucose and depressive symptoms among females became non-significant when individuals with diabetes were excluded, and this is consistent with the known comorbidity of diabetes and mental disorders. With high fasting plasma glucose levels characterising diabetes, it is suggested that the monitoring of depressive symptoms alongside traditional risk factors for diabetes is warranted, particularly given that all other relationships remained consistent with the larger sample.

Self-appraised diet quality was significantly associated with depressive symptoms for both males and females, independent of all other demographic and behavioural factors. While this measure was limited in that it remains unknown how closely self-appraised diet corresponded to actual dietary habits, this measure has previously been shown to correlate to a more comprehensive dietary index in a US representative sample [31]. The observed relationship was unsurprising given mounting evidence for a diet–depression relationship whereby individuals consuming foods considered unhealthy (e.g., processed foods with little nutritional value), more frequently report mental health problems compared to individuals with healthy diets [46,47]. As discussed, poor diet quality is also a key driver of poor glucose regulation [48,49,50]. Dietary strategies are regularly promoted as a therapeutic approach in diabetes and have benefits on glucose regulation and disease risk in non-diabetic populations [51]. Specifically, there are associations between greater intakes of whole-grain foods [52] as well as Mediterranean-style diet [53] and lower fasting glucose. Concurrently, there is consistent evidence supporting the importance of healthful, ‘traditional’ dietary patterns to the prevention of major depressive disorders [54,55]. In a recent meta-analysis, a ‘Western’ diet, characterised by highly-processed foods and low fruit and vegetable intakes, was associated with an increased risk of depression, whereas a traditional ‘healthful’ dietary pattern, comprising fruits, vegetables, whole grains, and few animal foods and was associated with decreased risk [56]. Further, in a recent randomised controlled trial, participants allocated to a 12-week dietary intervention following a Mediterranean-style diet showed significant reductions in depressive symptoms compared with those in the social support control group, with nearly one third of those in the dietary group achieving remission [57].

In the current study, women had higher BMI and lower fasting plasma glucose compared to men at baseline, and there were significant differences in the glucose-depression association between men and women. Our findings in women are supported by previous studies on gender differences in adipose tissue distribution and energy (glucose and fat) metabolism [58]. Specifically, women have a higher body fat percentage and insulin sensitivity, and the relationship between obesity and depression appears to be more pronounced in women [59,60]. Furthermore, gender differences have been identified in leading risk factors contributing to global disability-adjusted life years (DALYs) [17]. Smoking, high systolic blood pressure and high BMI emerged as the highest contributors to DALYs for men, and high systolic blood pressure, high body mass index and high fasting plasma glucose were the leading risk factors for women [17]. It has been suggested that the global increase of fasting plasma glucose is likely to be associated with global increases in BMI (significantly associated with depressive symptoms in this study) thus highlighting the complexity in risk factors and subsequent health outcomes for men and women. 

Beyond fasting plasma glucose and self-appraised diet quality, a number of variables were associated with increased levels of depressive symptoms including; level of socioeconomic disadvantage, higher BMI (females) and older age (males). Poorer mental health outcomes and increased prevalence of common mental disorders have been consistently shown in disadvantaged communities [61,62]. Such communities also experience poorer access to healthcare and preventive measures, low levels of health literacy, and other complex significant health and societal challenges compared to advantaged communities [63,64]. The findings here demonstrate the ongoing need for specific psychological support in communities experiencing disadvantage. The finding that increased BMI was associated with increased levels of depressive symptoms is also consistent with previous research [65], and as discussed above, could be due to adiposity-related physiological mechanisms. The bi-directional relationship between overweight/obesity and depression is also widely reported with sociological, psychological and cultural factors considered to drive this relationship [66]. Further, low mood, perceived stress and depressive symptoms have been associated with higher intakes of energy-dense, highly palatable (i.e., high-fat, high-sugar) foods, and lower intakes of fruits and vegetables [67]. While evidence supports the connection between emotion, food choice and craving, particularly in women [68], it remains unclear whether individuals with depression have poor long-term diet quality. Finally, the finding that older age among men, but not women, was significantly associated with depressive symptoms was of particular interest, and this holds potential for future age- and gender-related epidemiological research.

### Limitations

The cross-sectional design of this survey precludes conclusions on causality. While this study was strengthened by the combined analysis of biomedical, behavioural and psychological factors, many of the items required self-report thereby limiting the extent to which the data truly reflect health status. The self-appraised diet quality measure in particular was limited to an individual’s knowledge and perceptions of diet quality and given significant findings in the relationship with depressive symptoms, future research is required to comprehensively assess objectively measured dietary intake and mental health status. We cannot assume that self-appraised diet quality corresponded to actual dietary quality as this item was self-reported with no criteria except participants’ beliefs. However previous research has shown consistency between self-appraised diet quality and more comprehensive dietary index, and is widely accepted for population studies of this size and scale [31]. Further research is also planned to investigate the directionality of associations between glucose, lifestyle and depression, and thus extrapolate potential mechanisms at work at a population-level. Physical activity in particular has been shown to independently mediate the expression of both biomarkers and mental health symptoms, and was not adequately captured in this study. 

## 5. Conclusions

To our knowledge this is the first study to report the relationship between fasting plasma glucose and depressive symptoms, in a large US representative population of adults that adopted a biopsychosocial approach. Our findings indicate that glucose regulation and depressive symptoms were significantly associated among females, and that self-appraised diet quality was significantly related to depressive symptoms. Future longitudinal research should aim to examine directionality and the role of gender in these associations. Understanding the potential for shared underlying risk factors is an important area of research for the prevention and management of individuals living with depression, T2D, and indeed, comorbid conditions. This study is a preliminary, albeit critical, step in building the evidence base for comorbid health outcomes through modifiable risk factors. 

## Figures and Tables

**Table 1:** Participant characteristics.

Characteristics	Male ^a^ *n* = 1031 (48.9% Weighted)	Female ^a^ *n* = 1114 (51.1% Weighted)	Total ^a^ *n* = 2145
Age in years, *n* (%)			
18–24	137 (14.0)	144 (12.4)	281 (13.1)
25–34	159 (17.6)	170 (17.1)	329 (17.3)
35–44	165 (17.9)	191 (17.1)	356 (17.5)
45–54	171 (17.9)	168 (15.1)	339 (16.5)
55–64	172 (15.8)	206 (18.8)	378 (17.3)
65–74	128 (10.8)	140 (11.9)	268 (11.4)
75–80	99 (5.9)	95 (7.6)	194 (6.8)
Annual income USD, *n* (%)			
$0–19,999	232 (16.2)	269 (18.9)	501 (17.6)
$20,000–54,999	382 (33.8)	441 (36.1)	823 (35.0)
$55,000–74,999	110 (12.1)	121 (12.4)	231 (12.3)
$75,000–99,999	106 (12.6)	104 (11.4)	210 (12.0)
$100,000+	201 (25.3)	179 (21.0) *	380 (23.1)
Depressive symptoms			
Depressive symptoms *m* (SD) (0–27)	2.5 (3.8)	3.6 (4.4) *	3.1 (4.2)
Major depression ^1^ *n* (%)	71 (6.4)	127 (10.4) *	198 (8.4)
Glycaemic control			
Fasting plasma glucose (mmol/L) *m* (SE)	5.9 (1.7)	5.7 (1.7) *	5.8 (1.7)
Diabetes, *n* (%)	120 (9.4)	122 (10.0)	242 (9.7)
Weight status			
BMI, *m* (SD) kg/m^2^	28.5 (6.2)	29.7 (8.2) *	29.1 (7.3)
Lifestyle factors			
How healthy is diet? *n* (%)			
Excellent/very good	319 (29.3)	324 (31.7)	643 (30.5)
Good/fair	656 (65.9)	722 (62.4)	1378 (64.1)
Poor	56 (4.8)	68 (5.9)	124 (5.4)
Daily time spent sedentary *n* (%)			
<3 h	159 (14.9)	189 (16.2)	348 (15.6)
3–5 h	213 (19.9)	233 (20.5)	446 (20.2)
More than 5 h	659 (65.1)	692 (63.2)	1351 (64.2)

^a^ Sample sizes are unweighted and proportions/means are weighted to account for sampling strategy (proportions may not total to 100% due to rounding); ^1^ Major Depression is defined as score of equal to or greater than 10 out of 27, as recommended by PHQ-9 co-authors [26]; *m* = mean; SD = standard deviation; BMI = body mass index. * Significant (*p* < 0.05) difference between males and females.

**Table 2:** Unstandardized (*b*) and standardized (β) coefficients from linear regression analyses for depressive symptoms (DV) and fasting glucose (mmol/L) (IV), unadjusted and adjusted for age, gender, income, BMI, diet, time spent sedentary, weighted to account for sampling strategy.

Models	Males *n* = 1031				Females *n* = 1114			
	Unstandardized *b*	95% CI for *b*	β	*p*	Unstandardized *b*	95% CI for *b*	β	*p*
Model 1								
Fasting glucose (mmol/L)	0.08	−0.11, 0.26	0.03	0.420	0.24	0.05, 0.43	0.09	0.013
Model 2 (+demographics)								
Fasting glucose (mmol/L)	−0.02	−0.22, 0.18	−0.01	0.831	0.21	0.03, 0.38	0.08	0.021
+Age (years)	0.22	0.04, 0.39	0.10	0.015	0.01	−0.13, 0.15	0.00	0.926
+Household income (USD)	−0.44	−0.64, −0.24	−0.17	0.000	−0.55	−0.78, −0.32	−0.18	0.000
Model 3 (+weight status)								
Fasting glucose (mmol/L)	−0.06	−0.25, 0.14	−0.02	0.562	0.13	−0.04, 0.30	0.05	0.142
+Age	0.21	0.04, 0.39	0.10	0.015	0.01	−0.13, 0.15	0.01	0.855
+Household income	−0.44	−0.63, −0.24	−0.17	0.000	−0.51	−0.74, −0.28	−0.16	0.000
+BMI (kg/m^2^)	0.04	−0.01, 0.08	0.06	0.088	0.06	0.02, 0.09	0.11	0.003
Model 4 (+lifestyle factors)								
Fasting glucose (mmol/L)	−0.09	−0.29, 0.12	−0.04	0.405	0.11	−0.06, 0.28	0.04	0.211
+Age	0.25	0.07, 0.43	0.12	0.007	0.08	−0.06, 0.23	0.03	0.260
+Household income	−0.39	−0.58, −0.19	−0.15	0.000	−0.43	−0.66, −0.20	−0.14	0.000
+BMI	0.03	−0.01, 0.08	0.06	0.120	0.04	0.00, 0.07	0.07	0.045
+Self-appraised diet quality								
Poor	Ref.				Ref.			
Good/fair	−2.46	−4.14, −0.78	−0.31	0.004	−3.31	−4.77, −1.86	−0.36	0.000
Very good/excellent	−2.85	−4.58, −1.12	−0.34	0.001	−4.11	−5.64, −2.57	−0.43	0.000
+Daily sedentary time								
<3 h	Ref.				Ref.			
3–5 h	0.86	−0.19, 1.92	0.09	0.108	−0.78	−1.68, 0.12	−0.07	0.089
More than 5 h	0.38	−0.33, 1.09	0.05	0.294	0.10	−0.71, 0.91	0.01	0.810

*b* = unstandardized coefficient; *β* = standardised coefficient; 95% CI = 95% confidence interval; BMI = body mass index; bolding indicates significance *p* < 0.05.

## Supplementary Materials

The following are available online at www.mdpi.com/2072-6643/9/12/1330/s1, Table S1: Unstandardized (*b*) and standardized (*β*) coefficients from linear regression analyses of sub-group with doctor diagnosed diabetes individuals excluded, for depressive symptoms (DV) and fasting glucose (mmol/L) (IV), unadjusted and adjusted for age, gender, income, BMI, diet, time spent sedentary, weighted to account for sampling strategy.
