# Supplementary material for: Fasting Plasma Glucose, Self-Appraised Diet Quality and Depressive Symptoms: A US-Representative Cross-Sectional Study
Source: Nutrients. 2017 Dec 7;9(12):1330. doi: 10.3390/nu9121330 (PMC5748780; doi:10.3390/nu9121330)
Supplement: Supplementary file 1 [file nutrients-09-01330-s001.pdf]

Supplementary Table 1. Unstandardized (b) and standardized (β) coefficients from linear regression analyses of sub-group with doctor diagnosed diabetes individuals excluded, for depressive symptoms (DV) and fasting glucose (mmol/L) (IV), unadjusted and adjusted for age, gender, income, BMI, diet, time spent sedentary, weighted to account for sampling strategy

	**Males *n* = 911**				**Females *n* = 992**			
	Unstandardized *b*	95%CI for *b*	*β*	*p*	Unstandardized *b*	95%CI for *b*	*β*	*p*
Model 1								
Fasting glucose (mmol/L)	0.20	-0.24, 0.63	0.05	0.375	0.20	-0.09, 0.49	0.04	0.173
Model 2 (+ demographics)								
Fasting glucose (mmol/L)	0.04	-0.44, 0.52	0.01	0.865	0.10	-0.20, 0.41	0.02	0.507
+ Age (years)	0.21	0.01, 0.41	0.10	0.039	-0.03	-0.17, 0.12	-0.01	0.720
+ Household income (USD)	-0.46	-0.67, -0.25	-0.18	0.000	-0.55	-0.79, -0.31	-0.18	0.000
Model 3 (+ weight status)								
Fasting glucose (mmol/L)	-0.05	-0.52, 0.41	-0.01	0.823	-0.02	-0.32, 0.27	-0.00	0.868
+ Age	0.21	0.02, 0.41	0.10	0.035	-0.02	-0.16, 0.13	-0.01	0.828
+ Household income	-0.46	-0.67, -0.25	-0.18	0.000	-0.51	-0.75, -0.27	-0.17	0.000
+ BMI (kg/m^2)	0.04	-0.01, 0.09	0.07	0.088	0.05	0.01, 0.09	0.09	0.011
Model 4 (+ lifestyle factors)								
Fasting glucose (mmol/L)	-0.07	-0.53, 0.39	-0.02	0.754	-0.03	-0.31, 0.26	-0.00	0.859
+ Age	0.24	0.03, 0.44	0.11	0.022	0.04	-0.11, 0.19	0.02	0.605
+ Household income	-0.40	-0.61, -0.20	-0.16	0.000	-0.45	-0.70, -0.21	-0.15	0.000
+ BMI	0.04	-0.01, 0.09	0.06	0.112	0.03	-0.00, 0.07	0.06	0.082
+ Diet								
Poor	Ref.				Ref.			
Good/fair	-2.73	-4.59, -0.88	-0.34	0.004	-3.09	-4.63, -1.55	-0.35	0.000
Very good/excellent	-3.02	-4.92, -1.12	-0.36	0.002	-3.69	-5.31, -2.08	-0.41	0.000
+ Daily sedentary time								
<3 hours	Ref.				Ref.			
3 – 5 hours	0.95	-0.17, 2.06	0.10	0.095	-0.54	-1.46, 0.39	-0.05	0.254
More than 5 hours	0.32	-0.42, 1.06	0.04	0.399	0.22	-0.60, 1.04	0.02	0.602

b = unstandardized coefficient; *β* = standardised coefficient; 95%CI = 95% confidence interval; BMI= body mass index
